# Nursing undergraduates’ experience of participating in the Internet + Nursing Program and their spiritual education needs: A qualitative study from the perspective of the Neuman System Model

**DOI:** 10.12669/pjms.40.8.8822

**Published:** 2024-09

**Authors:** Ying Huang, Xiaotong Dong, Wei Wu, Haoran Zhang, Fei Qi

**Affiliations:** 1 Ying Huang Department of Nursing, Chengde Medical University, Chengde 067000, Hebei, China; 2 Xiaotong Dong Department of Nursing, Chengde Medical University, Chengde 067000, Hebei, China; 3 Wei Wu Department of Haemodialysis Unit, Affiliated Hospital of Chengde Medical College, Chengde, Hebei, 067000, China; 4 Haoran Zhang Department of Cardiology, Affiliated Hospital of Chengde Medical College, Chengde, Hebei, 067000, China; 5 Fei Qi Department of Nursing, Chengde Medical University, Chengde 067000, Hebei, China

**Keywords:** Neuman system model, Undergraduates, Spirituality, Spiritual education, Spiritual nursing, Internet

## Abstract

**Objective:**

This study aimed to explore the qualitative aspects of nursing undergraduates’ experience of participating in the Internet + nursing program and their spiritual education needs from the perspective of the Neuman system model.

**Methods:**

Using a descriptive qualitative study design, twelve full-time undergraduates at Chengde Medical University from June to July 2022 who had completed their clinical internship were interviewed one-on-one, and a purposive sampling method was adopted. Moreover, Colaizzi’s phenomenological method was employed to analyze the data and improve the themes.

**Results:**

In the Internet + nursing program, the lack of spiritual education for undergraduates can be summarized into the following four themes: lack of spiritual consciousness; lack of spiritual education and spiritual nursing knowledge; high demand for spiritual education; and cognition of the current situation of basic nursing education in China and thoughts on incorporating Internet-related technologies into future nursing education.

**Conclusion:**

At the school and hospital level, importance should be placed on the basic education of spirituality and psychological nursing, and appropriate measures should be taken to improve the knowledge level of spirituality and psychological nursing as well as the capacity of spiritual nursing, in order to ensure high-quality nursing services.

## INTRODUCTION

The Neuman system model defines each person’s system as a complex composed of psychological, physiological, social and spiritual components, in which psychological health is a major variable in maintaining personal stability.^1^ In the model, spirituality has a positive or negative impact on other variables (body, mind and society), which in turn affect the spirituality variable. To this end, an assessment of an individual’s spiritual cognition and spiritual needs will lead to a better understanding of health and optimal use of energy resources.^2^ Spiritual nursing is a nursing practice in which nursing staff, i.e., undergraduates and faculty, assess patients’ spiritual needs and then provide culturally appropriate nursing. They meet patients’ spiritual needs by engaging in caring, listening, and finding the meaning of life, enabling patients to achieve spiritual well-being. It is considered that whether undergraduates have received education about psychological health nursing is a crucial factor affecting their psychological health nursing skills.

However, undergraduates in China have limited access to psychological nursing education and psychological health education, resulting in their inability to meet patients’ psychological health needs when engaging in clinical practice.^3^ This raises a series of questions, such as how undergraduates view spirituality. Are they knowledgeable enough about spiritual nursing? Nursing undergraduates are currently ill-equipped to provide spiritual nursing services to clinical patients given the little spiritual education they have received. This is in stark contrast to their great need for spirituality and spiritual education. It is suggested that nursing educators should focus on designing spiritual education courses and integrating them into the teaching and practice of nursing undergraduates, so as to meet their spiritual education needs. By doing so, nursing undergraduates are equipped to render better spiritual nursing services to patients in their later clinical practice. Under the theoretical guidance of the Neuman system model, undergraduates who graduated from a domestic university were selected in this study to comprehend their understanding of spirituality and their needs for spiritual nursing education. By doing so, a theoretical basis can be provided for nursing educators and clinical nursing managers to pay attention to undergraduates’ spiritual cognition and spiritual nursing education. The theoretical basis is described in this manuscriptr.^4^

## METHODS

Guided by a qualitative phenomenological approach, respondents were interviewed and relevant data were collected using a semi-structured interview method. Twelve undergraduates from nursing undergraduates at Chengde Medical University from June to July 2022 was used the purposive sampling method to select for this study. Twelve undergraduates, including 10 females and two males, were interviewed. Their age ranged from 21 to 23, with an average age of (21.83±0.75) years (Table-I).

**Table-I:** General demographic information of respondents.

Serial No.	Gender	Age	Religious belief	Have they received spiritual education and where did it come from?	Internship location and hospital nature
1	Female	22	None	No	A Grade-A tertiary hospital in Shanghai
2	Female	21	None	No	A Grade-B tertiary hospital in Hebei Province
3	Female	21	None	No	A Grade-A tertiary hospital in Hebei Province
4	Male	22	None	No	A Grade-A tertiary hospital in Shandong Province
5	Female	23	None	No	A Grade-A tertiary specialized hospital in Tianjin
6	Female	22	None	No	A Grade-A tertiary hospital in Hebei Province
7	Female	21	None	No	A Grade-B tertiary hospital in Shanghai
8	Male	22	None	No	A Grade-A tertiary hospital in Beijing
9	Female	21	None	Rarely, contact during internship	A Grade-A tertiary hospital in Henan Province
10	Female	23	None	Rarely, contact during internship	A Grade-A tertiary hospital in Beijing
11	Female	22	None	No	A Grade-A tertiary hospital in Shanghai
12	Female	22	Yes, Catholicism	No	A Grade-A tertiary hospital in Hebei Province

### Ethical Approval:

The study was approved by the Institutional Ethics Committee of Chengde Medical University (No.: CYFYLL2018003; January 01, 2018), and written informed consent was obtained from all participants.

### Inclusion criteria:

- Undergraduates with clear language skills who voluntarily participated in this study.
- Full-time undergraduates.
- Senior undergraduates who have completed clinical practice for one year.
- Undergraduates who had a good willingness to cooperate with investigators.

### Exclusion criteria:

- Based on the principle of information saturation, data collection was stopped if no new themes emerged from the data analysis.

Using the Newman system model setting as a theoretical guide, a qualitative study was conducted using Colaizzi’s phenomenological methods on four aspects: “spiritual needs”, “spiritual cognition”, “school plans for spirituality and spiritual care” and “Internet + nursing program”. After drafting an interview schedule based on four aspects, the research team trained its members in qualitative interview techniques. Subsequently, pre-interviews were conducted with 12 nursing undergraduates with their written informed consent, and their privacy was protected.

To prevent the interviewees from thinking and preparing too much about the questions in advance, which will affect the results, the interview questions were not revealed to them before the interview, and a relaxed and pleasant atmosphere was kept as much as possible during the interview, so as to obtain their honest views. We contacted each interviewee before the interview and explained the purpose, method, importance and privacy measures of the interview in detail, and signed an informed consent form. Each interview lasted 30–45 minutes. During the interview, the behavior, tone changes, facial expressions and other nonverbal communication behaviors of undergraduates were observed in order to improve and adjust the interview schedule. The main points of the adapted syllabus are as follows:

- How do you understand “spirituality”?
- How do you understand spiritual care?
- How do you evaluate your psychological health?
- How do you provide spiritual care for clinical patients?
- What suggestions do you have for providing psychological health courses?
- What are your understanding and thoughts on participating in Internet-related work in the future?
- How much do you know about “Internet +” nursing? The specific content of the interview is as follows:
- What is spirituality?
- What is spiritual care?
- School perspectives on offering spirituality and spiritual nursing courses;
- Understanding of the current situation of undergraduate nursing in China and the participation of Internet-related technologies in future nursing practice.
- Understanding and thoughts on future participation in Internet-related work;
- Thoughts on the current situation of undergraduate nursing education in China and the future development of Internet-related technologies in the nursing field. When undergraduates made it clear in the interview that they knew nothing about spirituality, the investigators encouraged them to express their views in their own words, and asked follow-up questions only when appropriate.

### Data analysis:

Colaizzi’s phenomenological data analysis method was used to encode the data. The investigators listened carefully to the recordings and summarized them into text materials within 24 hour after the interview ended. They reread the text, extracted key statements, and coded recurring ideas. They then wrote a detailed incomplete description, sublimating thematic concepts and identifying similar ideas. Finally, they reappeared with the respondents until no new themes emerged, that is, the interview ended when data saturation was reached.

### Quality control:

Considering the different characteristics of the interviewees in terms of occupation and team role, the representativeness of the interviewees was fully considered, and appropriate adjustments were made to the way and content of questioning. Appropriate communication methods and question order were used to guide the interviewees’ thinking. Throughout the interview, an appropriate questioning tone was maintained without being overly judgmental and emotional. For the authenticity of information to avoid the Hawthorne effect, the investigators jointly identified appropriate interviewees during the data collection phase and were transcribed by one of the investigators while reviewed by the other. Once all interview data was compiled it was returned to the interviewees for review to ensure the reliability of the results. Valuable words were carefully screened out, considered in different interviewees’ backgrounds, and ambiguous words were reconfirmed with interviewees in the process of data collection. Data collection was completed after data saturation.

## RESULTS

By comparing the interview data of 12 interviewees repeatedly, four themes emerged: undergraduates’ lack of cognition of spirituality, lack of cognition of spiritual nursing, their desire for spirituality and spiritual nursing education, and their thoughts on integrating future nursing education into Internet-related technologies. The C-SCGS scores showed in Table-II.

**Table-II:** C-SCGS scores (n=251).

	Mean±standard deviation	Median (p25, p75)	MIN value	MAX value	Total score
Spiritual care characteristics	63.17±8.15	65 (60,66)	28	78	78
Spirituality and spiritual care definition	36.51±5.61	37 (32,40)	20	48	48
Spiritual cognition	24.18±3.48	25 (23,26)	6	30	30
Spirituality and spiritual care value	37.51±5.17	39 (34,40)	17	48	48
Total score	161.37±19.53	166 (151,170)	95	204	204

Although the 12 subjects in this study were undergraduates who were about to graduate after four years of university study and one year of clinical practice, most of them could neither give a clear explanation of the word ‘spirituality’ nor have any knowledge about the word ‘spirituality’ on the Internet. When asked to explain the meaning of the word, they were unable to come up with any definition.^5^,^6^ *Interviewer:* This interview is about spiritual cognition and spiritual education needs. I have a few questions to communicate with you. Do you know anything about spirituality? *Interviewee 1:* In my opinion, spirituality means not being troubled by external things, and being able to have a new understanding and view after breaking through the troubles in life.

Undergraduates were able to make their own explanations of spirituality, but they did not understand it deeply enough. *Interviewee 11:* I think spirituality is an id idea. You may have a certain view on a matter before you receive education in this aspect, but after your parents’ education and some influence from society, your view on this matter may be affected. Therefore, I think spirituality may be a kind of id view. Literally, spirituality should describe a person’s intelligence and wisdom, but in essence, it should have a deeper meaning.^7^,^8^

When asked about their spiritual health, most undergraduates said they knew nothing about it. *Interviewer:* This interview is about two aspects of spiritual cognition and spiritual education needs. I want to ask you about your understanding of spirituality. In the previous study process, we have also learned more about nursing, physical, psychological, and social aspects, but now spirituality, the deepest knowledge, has been derived. These four aspects of health come together into holistic patient care. So, what do you understand about spirituality? *Interviewee 2:* In the hospital, nurses give patients a kind of psychological support, and guidance or listen to their demands when they are sick or facing death, and then help them rebuild their confidence in their future life and overcome their illness.

In terms of spiritual education, most undergraduates believe that schools should offer courses on spirituality and spiritual care.^9^,^10^ *Interviewer:* This interview is about spiritual cognition, spiritual education and spiritual needs. Let’s talk about the first point, spirituality, which has just been explained. Health is divided into four aspects: physical, psychological, social and spiritual. We have learned the first three aspects in our undergraduate study and learned a lot, but we have relatively little involvement in spirituality. So, I want to ask you how you understand spirituality and how you know about spirituality. *Interviewee 5:* No matter what stage of the disease a person is in, the disease will have some psychological impact on him, causing him to be more fearful and lose some goals for the future of life. Therefore, spiritual care can be used to help him reshape his future life.

Many undergraduates expect to use their knowledge and skills to help patients and soothe their wounded hearts, thereby alleviating their pain.^11^ *Interviewer:* Today we are talking about some problems with spiritual cognition and spiritual education needs. We all know that health is a person’s physical, psychological, social and spiritual health. We have known and learned more about the first three levels in our usual studies, but we have relatively little awareness of spirituality. So how do you understand spirituality? How do you know about spirituality? *Interviewee 6:* Spirituality means paying attention to one’s inner feelings, following one’s own values, and then seeking what one really wants in one’s heart and surviving on one’s own.

Most interviewees’ understanding of “Internet + nursing” is limited to the mass internet combined with various network platforms or apps (such as WeChat). When answering the question about “Internet + nursing”, most of them answered that they knew too little about it. The investigators said, “Every interview has an obvious sense of limitation, and when discussing Internet-related issues, it is often superficial.”

At present, undergraduate education is still limited to books on medical and nursing knowledge, which are updated slowly and lack cutting-edge knowledge dissemination on the Internet. Undergraduates usually go to the library to consult relevant articles or find relevant information on the Internet.^12^,^13^ *Interviewer:* Today’s interview is about spiritual education. After studying specialized courses, you probably know that health is a whole. Therefore, we would like to first understand how you came to know about spirituality. What do you think is spirituality? *Interviewee 7:* Spirituality is a kind of spiritual sustenance because its definition contains the meaning, goal and value of life. If a person wants to reach a certain height and then works hard in this direction, the guidance in this direction is called spirituality.

## DISCUSSION

For the first time, we focused on Chinese undergraduates’ cognition of spirituality and their needs for spiritual education through a qualitative study based on the Newman system model.^14^ The results show that although undergraduates report that they have “heard” about spirituality in their undergraduate education and clinical practice, most of them have misunderstandings or prejudices about spirituality, or they don’t know that this is consistent with the current university education and clinical practice. It is consistent with the current university education and clinical practice, and also with the relevant research results at home and abroad. Domestic literature shows that the concept of spirituality has not been widely used in our lives and clinical work. The Newman system model shows that spirituality is a relatively new concept in terms of psychological, physiological, physical and social variables, and people may or may not realize the existence of spirituality.

In severe cases, they fail to correctly identify patients with psychological health needs in future clinical practice and are therefore unable to provide them with spiritual care. It can be seen that providing spiritual education for undergraduates and ensuring that they have a correct and clear understanding of spirituality can ensure that they form a healthy spiritual understanding.^15^,^16^ The results of this study show that although they are about to graduate and enter clinical nursing after completing one year’s clinical practice, most undergraduates have not received any formal courses and training related to mental health nursing teaching during their school and practice.

In other words, undergraduates perform poorly in psychological health nursing abilities and need to further improve it.^4^ As we all know, undergraduates, as future nursing professionals, are the health professionals who are most likely to come into contact with patients and are also the main providers of mental health care. Their psychological healthcare skills are directly related to the psychological health of clinical patients. Most undergraduates hope to receive education related to spirituality in school and clinical practice.^17^

Although most undergraduates don’t have a clear understanding of spirituality, they put forward their own suggestions for schools and hospitals to carry out spiritual education and spiritual care teaching. For example, some undergraduates suggested sharing clinical cases and clinical experiences.^18^ At present, spiritual education and spiritual care education have not been included in the unified curriculum in China. Explore the traditional culture of our country, and combine China’s traditional philosophy, life education, health preservation, traditional medicine, humanistic care and other factors to form a mental health care service model with Chinese characteristics.^19^ In clinical practice, we should pay attention to finding the weak links in the process of psychological care for patients, formulate scientific and reasonable psychological education and psychological care education implementation plans, improve the psychological care ability of undergraduates, and provide effective methods for psychological care for patients after they enter their jobs.^20^

### Limitations:

Although the subjects are interns in many hospitals across the country, they are only undergraduates in one hospital, so the research results may not be more representative. It is suggested that the sample sources can be expanded in future research. In addition, given the small sample size of this qualitative study, it is necessary to further explore the current spiritual cognition of undergraduates and trainees and their future needs for spiritual care education in order to improve a more reliable foundation for the development of spiritual care education.

## CONCLUSIONS

To sum up, we interviewed 12 undergraduates who formally entered the clinical nursing work in this study to understand their cognition and practical experience of spirituality and their needs for spiritual nursing education. It was found that undergraduates’ cognition of spirituality was poor and their needs for spiritual nursing education were great, indicating that nursing educators and clinical nursing leaders should pay attention to the spiritual education and spiritual nursing education of undergraduates during their school and internship.

### Authors’ Contributions:

**YH** and **FQ:** Carried out the studies, participated in collecting data, drafted the manuscript, are responsible and accountable for the accuracy or integrity of the work.

**XD:** Performed the statistical analysis and participated in its design.

**WW** and **HZ:** Performed the statistical analysis, interpretation of data and participated in its design.

All authors have read and approved the final manuscript.
